# Adjustable-tip needles versus fixed-tip needles in radiofrequency ablation of symptomatic benign thyroid nodules: a single-center Italian experience

**DOI:** 10.1007/s40477-024-00926-4

**Published:** 2024-06-22

**Authors:** Mattia Rossi, Letizia Meomartino, Loredana Pagano, Giulia Follini, Sara Garberoglio, Mauro Maccario, Ruth Rossetto Giaccherino, Roberto Garberoglio

**Affiliations:** 1 Endocrinology, Diabetology and Metabolism, Department of Medical Sciences, University of Turin, Corso Dogliotti n.14, 10126 Turin, Italy; 2 Thyroid Multidisciplinary Center, Humanitas Cellini, Turin, Italy

**Keywords:** Adjustable-tip needle, Benign thyroid nodule, Radiofrequency ablation, Minimally-invasive thyroid treatment

## Abstract

**Purpose:**

In this retrospective, observational study we aim to compare the outcomes of the RFA treatment of benign thyroid nodules, carried out respectively with the standard fixed-needle approach (FTN) and the adjustable-tip needle technique (ATN), considered a more tailored, quicker and easier technical approach.

**Methods:**

We enrolled 36 patients who underwent RFA treatment of symptomatic, benign, thyroid nodule, 18 with the ATN and 18 with the FTN approach, respectively. Data about absolute volume reduction, volume reduction rate (VRR) and success rate (defined as VRR ≥ 50%), after 1, 3 and 6 months of follow-up were compared.

**Results:**

Our study suggested no substantial difference between the approaches, up to 6 months of follow-up, both in terms of absolute reduction (*p* = 0.27) and VRR (*p* = 0.14). These results were confirmed when the success rates, both in terms of 50%-reduction (*p* = 0.12) and absolute reduction (*p* = 0.42), was considered. Only at the 6-month evaluation, the FTN procedure showed a better success rate, yet without statistical significance (88.9% vs. 61.1%, *p* = 0.12). No difference emerged both in terms of patients’ satisfaction and safety.

**Conclusion:**

Our small experience suggested no substantial difference between ATN and FTN, in terms of outcomes. On the other hand, ATN was considered to be more straightforward and could consequently allow for a shorter operator learning curve.

## Background

Up to 60% of adults in the general population have one or more thyroid nodules [1]. However, most thyroid nodules are benign, with cancer occurring in approximately 7–15% of patients [1, 2].

Surgery has traditionally been the standard of care for symptomatic or cosmetically disfiguring thyroid nodules [2, 3], but in the past few years, minimally invasive techniques (MITTs), such as radiofrequency ablation (RFA), ethanol ablation (EA), laser ablation (LA), high-intensity focused ultrasound (HIFU) and microwave ablation (MWA) [2], have gained popularity as alternatives to surgery for the treatment of benign symptomatic thyroid nodules [4].

In particular, RFA has been accepted as a safe and effective treatment modality [4–6] and has largely replaced surgery for the management of symptomatic benign thyroid lesions.

The traditional approach, which has reached the widest employment among experts, involves an electrode with a fixed active tip (FTN), with a moving-shot (or “multiple overlapping shot”) technique, based on sequential repositioning of the needle [5, 7, 8].

However, another approach based on an electrode equipped with an adjustable active tip (ATN) has recently gained popularity in the treatment of thyroid nodules. This kind of electrode allows customizable volumes of necrosis to be obtained and a larger portion of the nodule to be treated per single repositioning, reducing the number of movements of the needle and leading to a quicker and technically easier procedure, thus eventually shortening the learning curve of practitioners. The ATN was originally designed for hepatic tumors, and a large body of literature exists on that specific field of application, both regarding animal ex vivo and in vivo livers [9], and clinical employment in human patients [10–12]. However, very few studies exist describing this approach when considering thyroid nodule ablation [13, 14], even if several experiences have been rumored among experts.

The aim of this study was to compare the two approaches in terms of volume reduction and success rate.

## Materials and methods

### Population

This retrospective observational study included 36 patients referred to our Centre (“Città della Salute e della Scienza” University Hospital in Turin) for RFA of symptomatic benign thyroid nodules from October 2019 to December 2022.

Our population was divided into 2 groups:

- *ATN Group:* 18 patients who underwent RFA treatment using the electrode equipped with an adjustable active tip between October 2021 and December 2022.
- *FTN Group:* 18 patients with comparable ultrasound nodule features who underwent RFA using the fixed active tip needle between October 2019 and February 2022.

### Inclusion and exclusion criteria

The eligibility criteria for inclusion in the study were as follows:

- At least 18 years of age.
- Presentation with a single-nodule disease or a dominant and clearly detectable nodule in a multinodular goiter.
- Symptoms or cosmetic issues related to thyroid nodules.
- Serum calcitonin in the sex-specific range of normality according to our laboratory kit.
- Cytologically confirmed benign nodule.

Thyroid function does not appear among the indications for treatment; therefore, patients were enrolled regardless of their TSH level or concomitant use of l-thyroxine.

Patients who were pregnant, had suspected or cytologically proven malignancies or had confirmed coagulation disorders were excluded from treatment.

### Pre-procedural evaluation

All patients underwent, before the procedure:

- *Clinical evaluation:* we categorized symptom and cosmetic scores as defined in a previous consensus statement [15]. Subjective compression symptoms were assessed on a score from 0 to 10 (where 0 indicates the absence of nodule-related symptoms; 10 indicates the maximum tolerable discomfort) assigned by the patients themselves; the cosmetic assessment was performed based on a four-point scale recorded by the physician (1 = absence of palpable mass; 2 = palpable but not visible mass; 3 = cosmetic problem during swallowing alone; 4 = easily identifiable visible mass).
- *Biochemical evaluation*: thyroid stimulating hormone (TSH), serum free thyroxine (fT4), calcitonin, complete blood count, coagulation tests, dibucaine number and cholinesterase, hepatic and renal functions.
- *Ultrasound (US) examination:* using an Esaote MyLab Twice real-time US system with a linear multifrequency (7–14 MHz) probe to evaluate the following parameters: diameter (anteroposterior, transverse, and longitudinal) of each thyroid nodule, measured in millimeters (mm); the nodular volume, expressed in milliliters (mL), calculated by the ultrasound machine with the “ellipsoid volume formula” (length × width × depth × 0.524); the nodular echo-structure (solid when the fluid component was absent or < 10%; microcystic if the fluid component was 10–50% of the total volume; macrocystic when the fluid component affected 50–90% of the total volume); the echoic pattern (hypoechoic, isoechoic, or hyperechoic compared to the adjacent strap muscles of the neck); vascularization, assessed by eco-color Doppler examination (perinodular or endonodular vascularization); the presence or absence of calcifications; and the elastonographic pattern, evaluated by qualitative elastography (strain elastography) as per Rago et al. classification (patterns 1 and 2 as soft, pattern 3 as intermediate and patterns 4 and 5 as hard) [16].
- *Two separate ultrasound-guided fine-needle aspiration* (FNA) *biopsies,* to assess the benign nature of the nodule (class TIR 2 s. SIAPEC-IAP [17]).
- Baseline electrocardiogram.
- *Vocal cord function assessment,* performed by an otorhinolaryngologist before the ablation procedure.

### Procedures

RFA procedures were carried out on a day-hospital regimen after patients provided written informed consent. All procedures were performed by 2 operators with > 10 years of experience.

RFA was performed with the patient in the supine position with mild neck extension. Patients underwent treatment in a state of conscious sedation, and puncture-site anesthesia was performed with approximately 10 cc of ropivacaine (0.75%) injected around the thyroid gland, always with ultrasound guidance.

The FTN approach involved the use of an 18-gauge, 7 or 10 cm long, internally cooled electrode with a 10 mm active tip (RFT(S) Tip/RFTP(S) RF Medical Co. Ltd.) connected to a radiofrequency generator (Mygen M-3004). The electrode tip was initially placed in the deepest and most remote portion of the nodule, and then repeatedly repositioned, moving it backward toward the most superficial and accessible portion of the nodule, following the principle of the moving-shot technique. Before the procedure, the target nodule was divided into multiple predicted ablation units, and the session ended when all units were treated.

The ATN approach was characterized by the use of a 16-gauge electrode equipped with a 0.5–2.5 cm long adjustable active tip [RF VCT 10 XXB Versan ©] connected to the same radio frequency generator, with a mean exposure of 1.5 cm.

The needle was inserted freehand under ultrasound guidance, identifying two functional plans that allowed to achieve the widest necrosis area. In fact, the substantial difference between this method and the FTN method is that the exposed needle is inserted in the center of an ideal ellipse, and the number of repositionings is smaller.

For all patients, a trans-isthmic approach was implemented: the electrode was inserted under ultrasound guidance through the isthmus into the target nodule, thus allowing a better view of the electrode position and avoiding damage to the critical surrounding structures [6].

Both procedures were constantly monitored under real-time, B-mode ultrasound assessment to evaluate the correct positioning of the electrode within the lesion to be treated.

The electrode was cooled with refrigerated saline solution (approximately 5 °C), preventing the tissues from reaching a temperature higher than 100 °C, to avoid carbonization, and allowing a wide and homogenous ablation area.

During the planning phase, before each procedure, the distance between the nodule and the capsule and the presence of distinctive supplying vessels were assessed to establish the need for additional maneuvers such as hydrodissection and vessel ablation.

The applied power and energy, and the actual time of treatment were recorded at the end of each session. This allowed for later calculation of indirect indexes like delivered energy (i.e. total energy/basal volume) and volume reduction per kJ, i.e. (basal volume − final volume)/(total energy (J)/1000).

Contrast-enhanced ultrasound (CEUS) with SonoVue (Bracco, Milan, Italy) was performed at the beginning and just before the end of each procedure to evaluate nodule vascularization and thus identify the extent of the necrotic area and possible residual nodular portions that needed further treatment. was selected as the contrast agent. CEUS enhancement was evaluated qualitatively and compared with that of the surrounding parenchyma. Transient and complete hyperecogenicity of the target nodule, linked to the vapors generated during the procedure, and the absence of intralesional vascularization represented the end of the procedure.

Then, the electrode was extracted, and a final US evaluation was performed to exclude intra- or extra-nodular complications. The patients were kept in osbervation for at least 3 h and finally discharged, with oral analgesic therapy in selected cases. Each patient was advised to seek immediate referral to our institution if they experienced any unusual symptoms that might suggest the development of minor or major complications. Hematoma, superficial skin burns, transient voice change, alteration of thyroid function, pain unresponsive to paracetamol, nodule rupture requiring conservative treatment were defined as minor complications, while permanent voice change, full-thickness skin burns, nodule rupture requiring invasive treatment, and tracheal injury were considered among the major complications. Finally, cough, mild pain responsive to paracetamol and vasovagal symptoms resolving before discharge were considered common side effects.

### Follow-up

After RFA, patients underwent US and clinical evaluations at 1, 3 and 6 months. During each follow-up, the treated nodule’s volume and volume-reduction rate (VRR), based on the formula: VRR = [(initial volume − final volume) × 100]/initial volume, were measured [18].

Therapeutic success was defined as a > 50% VRR. To compare success in terms of absolute volume reduction, a threshold of 7 mL was chosen because it represents the baseline volume of the smallest nodule in the population and thus the largest reduction possible for all nodules. The therapeutic success rate was defined as the percentage of successfully treated nodules [19].

Signs and symptoms that might suggest any procedural complications were also investigated at each step of the follow-up.

### Statistical analysis

The statistical analysis was carried out using the application software R [R Core Team (2021)]. A *p* value < 0.05 was considered to indicate statistical significance for all the statistical analyses. Continuous variables are reported as medians and interquartile ranges (IQR), and categorical variables are reported as numbers of observations and rates. Fisher’s exact test was used to compare categorical variables, while the Wilcoxon test was used for continuous variables. Finally, a multivariate logistic regression model was performed to rule out possible confounders.

## Results

### Description of the population

Our study involved 36 subjects: 28 (78%) women and 8 (22%) men. The median age was 54.5 years (50.0–59.5 years). The median pretreatment compression score was 5.0 (3.75–6.0). The median pretreatment aesthetic score was 4 (4–4). The median volume before the procedures was 19.75 mL (19.75–30.75 mL). The two approaches showed several technical differences: ATN showed significantly greater total and delivered energies [75,600 (63,000—103,950) J, *p* =  < 0.01 and 4350 (2581–5331) J, *p* =  < 0.01, respectively] but reached a smaller volume reduction for each kJ of energy [0.12 (0.07–0.25) cc/kJ, *p* = 0.01]. Finally, the median duration of the FTN procedure was 16 (11–17) min, *p* = 0.04 (Table 1).

**Table 1:** Description of the study population, split according to the implemented approach

	ATN (*n* = 18)	FTN (*n* = 18)	*p* value
Age (years)	53 (50.50–57.5)	57 (50–60.5)	0.40
Aesthetic score (points)	4 (3.25–4)	4 (4–4)	0.07
Compressive score (points)	6 (3.25–6)	5 (5–6.75)	0.95
Baseline TSH (mUI/L)	1.4 (1.4–1.92)	1.1 (1.1–1.60)	0.06
Baseline volume (mL)	21.3 (21.25–33.725)	19 (19–30.25)	0.95
Total energy (J)	75,600 (63,000–103,950)	50,408 (37,219–57,083)	< 0.01
Delivered energy (J/mL)	4350 (2581–5331)	2359.2 (1837.5–3002.6)	< 0.01
Volume reduction per kJ (cc/kJ)^a^	0.12 (0.07–0.25)	0.26 (0.22–0.29)	0.01
Ablation time (min)	19 (15–25)	16 (11–17)	0.04
Echo-structure			1.00
Solid	11 (61%)	10 (55.6%)	
Microcystic	2 (11%)	2 (11%)	
Macrocystic	5 (27.8%)	6 (33.3%)	
Echoic pattern			0.01
Isoechoic	14 (77.8%)	6 (33.3%)	
Hypoechoi**c**	4 (22.2%)	12 (66.7%)	
Vascularization			0.37
Perinodular	12 (66.7%)	10 (55.6%)	
Endonodular	6 (33.3%)	8 (44.4%)	
Elastosonography			0.22
Elastic	0 (0%)	1 (16.7%)	
Intermediate	9 (50%)	9 (50%)	
Hard	9 (50%)	6 (33.3%)	
Location			0.37
Right lobe	12 (66.7%)	10 (55.6%)	
Left lobe	6 (33.3%)	8 (44.4%)	

Continuous variables are expressed as medians and interquartile ranges, while categorical variables are expressed as numbers of observations and rates. The *p* values for comparison are outlined for each feature. Fisher’s exact test was used for categorical variables, while the Wilcoxon test was used for continuous variables

*ATN* adjustable-tip needle, *FTN* fixed-tip needle, *IQR* interquartile range, *TSH* thyroid-stimulating hormone

^a^Volume reduction per kJ: (Basal volume − Final volume)/(Total Energy/1000)

### Volumetric reduction

No difference in the VRR was found to be significant at 1, 3 or 6 months (*p* = 0.94, *p* = 0.85 and *p* = 0.27, respectively). ATN reported a slightly lower, but not significant, reduction at the 6-month follow-up (10.55 mL vs. 12.5 mL, *p* = 0.27) (Table 2A).

**Table 2:** (A) Absolute volumetric reduction (mL) at 1-, 3- and 6-month follow-ups, expressed as median and interquartile range; (B) volume reduction rate (%) at 1-, 3- and 6-month follow-ups, expressed as median and interquartile range

	ATN (*n* = 18)	FTN (*n* = 18)	*p* value
A	Absolute volumetric reduction (mL)		
1 month	6.0 (3.275–7.850)	5.9 (4.7–8.0)	0.94
3 months	8.90 (6.20–14.12)	9.4 (7.75–16.48)	0.85
6 months	10.55 (6.175–16.125)	12.5 (9.65–16.02)	0.27
B	Volume reduction rate (%)		
1 month	33.50 (16.75–55.75)	29.50 (25.25–46.25)	0.67
3 months	55.50 (30.25–63.75)	52.00 (45.50–57.50)	0.80
6 months	54.50 (39.00–72.75)	63.50 (55.00–75.50)	0.14

p values were derived from the Wilcoxon test

*ATN* adjustable-tip needle, *FTN* fixed-tip needle

The same was observed in terms of the VRR. No significant difference was detected at 1, 3 and 6 months (*p* = 0.67, *p* = 0.80, *p* = 0.14, respectively), even though a slightly lower, yet not significant, reduction rate was detected in the ATN group at the 6-month follow-up (54.50 vs. 63.50%, *p* = 0.14) (Table 2B).

### Success rate

Evaluating success rate:

- When considering a reduction of at least 50%, only slight and nonsignificant differences were observed between the FTN and ATN groups after 1 and 3 months (respectively, 17% vs. 33%, *p* = 0.44 and 67% vs. 56%, *p* = 0.73). After 6 months, however, a 50% reduction in baseline volume was reached in 88.9% of patients in the FTN group versus only 61.1% in the ATN group. Even in this scenario, only a tendency toward statistical significance was observed (*p* = 0.12) **(**Table 3A**)**.
- We did not observe any difference in the percentage of patients who achieved a volumetric reduction of at least 7 mL after 1 or 3 months (39% vs. 33%, *p* = 0.72, and 77.8% vs. 72.2%, *p* = 0.70, respectively, for FTN and ATN). Interestingly, after 6 months, 83% of the FTN patients and 72% of the ATN patients achieved a 7 mL reduction, still without reaching statistical significance (*p* = 0.42) **(**Table 3B**)**.

**Table 3:** (A) Rates of volumetric reduction success (endpoint: ≥ 50%) at 1-, 3- and 6-month follow-ups, expressed as numbers and rates; (B) rates of volumetric reduction success (endpoint: ≥ 7 mL) at 1-, 3- and 6-month follow-ups, expressed as numbers and rates.

	ATN (*n* = 18)	FTN (*n* = 18)	*p* value
A	Success rate (endpoint: ≥ 50%)		
1 month	6/18 (33.3%)	3/18 (16.7%)	0.44
3 months	10/18 (55.6%)	12/18 (66.7%)	0.73
6 months	11/18 (61.1%)	16/18 (88.9%)	0.12
B	Success rate (endpoint: ≥ 7 mL)		
1 month	6/18 (33.3%)	7/18 (38.9%)	0.72
3 months	13/18 (72.2%)	14/18 (77.8%)	0.70
6 months	13/18 (72.2%)	15/18 (83.3%)	0.42

p values were derived from Fisher’s exact test

*ATN* adjustable-tip needle, *FTN* fixed-tip needle

Finally, we evaluated the influence of possible confounders on the success rate of a reduction of at least 50% with a multivariate logistic model. No feature showed statistical significance, not even for the different approaches (Table 4).

**Table 4:** The outcome of the multivariate logistic regression model used to assess the influence of possible confounders

Variables	VRR > 50% at 6 months (yes = 1, no = 0)		
	OR	95% CI	*p* value
Approach (ATN = 1, FTN = 0)	0.222	0.019–2.026	0.189
Delivered energy (J/mL)	0.999	0.998–1.001	0.141
Ablation time (min)	0.947	0.823–1.074	0.406
Hypoechogenicity (yes = 1, no = 0)	6.414	0.658–124.464	0.147

*VRR* volume reduction rate, *OR* odds-ratio, *CI* confidence interval, *ATN* adjustable-tip needle, *FTN* fixed-tip needle, *TSH* thyroid stimulating hormone

### Complications

No major or minor complications were reported at any step of the follow-up. Only common side effects, specifically pain (11.1% vs. 16.7%, *p* = 1.0) and vasovagal symptoms (5.6% vs. 5.6%, *p* = 1.0), were occasionally observed on the day of the procedure and easily resolved spontaneously or with paracetamol. However, no difference could be detected between the approaches (Table 5).

**Table 5:** Complications reported after the procedure and during follow-up

	ATN (*n* = 18)	FTN (*n* = 18)	*p* value
Major complications	0 (0%)	0 (0%)	-
Minor complications	0 (0%)	0 (0%)	-
Common side effects			
Paracetamol-responsive pain	2 (11.1%)	3 (16.7%)	1.0
Vasovagal symptoms	1 (5.6%)	1 (5.6%)	1.0
Cough	0 (0%)	0 (0%)	-

Minor complications included hematoma, superficial skin burns, transient voice changes, alterations in thyroid function, pain unresponsive to paracetamol, and nodule rupture requiring conservative treatment. Major complications included permanent voice change, full-thickness skin burns, nodule rupture requiring invasive treatment, and tracheal injury. Common side effects included cough, mild pain responsive to paracetamol and vasovagal symptoms resolving before discharge. *p* values were derived from Fisher’s exact test.

*ATN* adjustable-tip needle, *FTN* fixed-tip needle

## Discussion

In this retrospective observational study, we aimed to compare two different approaches to RFA for the treatment of benign thyroid nodules: the traditional moving-shot approach with a fixed active tip (FTN) versus the recently introduced adjustable active tip needles (ATN), which are believed to allow the treatment of a larger and more personalized portion of the nodule, reducing the need for needle repositioning and leading to a quicker and technically easier procedure, thus eventually shortening the learning curve of young practitioners.

Our study suggested no substantial difference between the two approaches, up to 6 months of follow-up, both in terms of absolute reduction and VRR. These results were confirmed when the success rates, both in terms of 50% reduction and absolute reduction, were considered. The VRR, i.e., the main indicator of outcome, in our population was in line with most reports in the general literature on the topic. More specifically, the 6-month median VRR reached with the FTN was 64%, which was confirmed in a recent large meta-analysis during a comparable follow-up [20]. No 6-month VRR was reported in the two studies analyzing ATN on thyroid nodules in humans [13]. Lee et al., in their preliminary smaller noncompared analysis, reported a reduction of 68.3% 12 months after the procedure, which, given the acknowledged further reduction expected between the sixth and the twelfth month (approximately 10% [21], could be speculated to be comparable with our data (54% at 6 months). Conversely, in their wider and compared analysis, Shin et al. reported a larger VRR (83.3%) in a subgroup of patients followed for a general, less-than-2-year period [14].

Only at the 6-month evaluation, the FTN procedure showed a feeble tendency toward a better outcome, which was more evident when the 50% success rate was considered. We initially hypothesized that this result could suggest that a real difference could emerge on a longer follow-up, and be consistent with a comparable initial VRR but a smaller ablation ratio (i.e., the proportion of the ablated volume to the total volume of the nodule [22]). Consequently, the initial substantial success of the procedure, both in terms of objective reduction and patient satisfaction, could have been thwarted by a subsequent tendency toward regrowth and the need for new intervention [23]. However, these aspects could not be analyzed in our evaluation, given the relatively short follow-up time.

Fortunately, the substantial long-distance effectiveness of ATN and its noninferiority against FTN were proven, even during a longer-than-2-year follow-up (89.7% vs. 94.0%, *p* = 0.167, *n* = 26 vs. *n* = 60, respectively) in a comparably large subpopulation of ATN [14].

The two treatment groups were selected from a homogeneous population, as no substantial difference was found in terms of baseline volume or nodule composition.

Considering procedural features, with the ATN, we were able to convey to the treated tissue a larger amount of energy, both total and delivered (i.e., the rate between the total released energy and the volume of the nodule) [24], yet reaching a smaller volume reduction per conveyed kJ. The delivered energy was correctly greater than 2670 J/mL, which is believed to be the threshold for optimizing success [25]. On the other hand, the ATN treatments took longer (19 vs. 16 min, *p* = 0.04). A median variation of 3 min surely does not constitute an element of great clinical significance for the procedure; nevertheless, it stands on the opposite of one would expect, given the supposed ease of implementation of this approach. Such evidence could suggest that a larger exposed tip allows simultaneous treatment of a greater nodule volume but still requires a longer time to fully convey its effect.

In their experience[13], Lee et al. reported a comparable and even longer duration (22.0 ± 8.9 min) but a greater amount of conveyed energy (95,172.3 ± 56,533.3 J), probably because of the consistent treatment of larger nodules (29.6 ± 6.3 mL) [13]. Conversely, Shin et al. reported a substantially smaller total energy (29.2 kJ, *p* = 0.069), a larger ablated volume/energy (0.3 cc/kJ, *p* =  < 0.001) and a shorter total time (627 s, *p* = 0.009) in their ATN subgroup, compared both to their FTN subgroup and our ATN subgroup, thus increasing the value of ATN effectiveness [14].

In this small sample, no major or minor complications followed any procedure, and common side effects were reported occasionally but promptly resolved spontaneously or after drug treatment. Even Lee et al. did not report any major complications and reported only 3 cases of minor complications (hematoma in two patients and a skin burn in one patient) with ATN, which resulted in an overall rate of 14.3% in their similarly small population [13]. Moreover, similar results were reported in their wider population by Shin et al., who reported no major immediate complications but only side effects and immediate minor complications (radiating pain and hematoma), without significant differences between the two electrodes (6.7% vs. 2.2%, *p* = 0.167, respectively for ATN and FTN) [14]. In general, RFA is widely considered a safe technique, and complications are strictly dependent on the experience of the center and the operator. The rate of major complications has been described in several large meta-analyses to be approximately 1–2%, while the rate of minor complications varies between 1 and 17%, with hematomas being the most frequent when excluding so-called side effects [26]. Consequently, the ATN complication rate does not seem to differ substantially from what is generally reported for RFA.

As a counterpart of being safe and efficient, these techniques have a learning curve. Several studies have highlighted how 40–60 procedures are needed to achieve adequate VRRs and symptomatic/cosmetic relief [27–31]. However, far more practice grants a more complete and stable result, being correlated with a better ablation ratio and fewer complications [28, 32].

The two experienced operators at our center, responsible for the treatment of our patients, when questioned about this small experience, reported that, due to the slightly larger diameter, the ATN led to simpler localization. Moreover, the mean need for needle repositioning was two times per procedure, and often a procedure was completed with no need for repositioning at all. Therefore, it can be hypothesized that the ATN technique may require less training than the FTN technique, but these data need to be confirmed with larger studies.

From a clinical point of view, more than 90% of patients reported complete resolution of compressive symptoms, irrespective of the implemented approach. Our study’s main limitations include its observational and retrospective nature and the unblinded selection of control patients, which are thought to reduce the weight of confounders such as baseline volume but could have resulted in selection biases. Moreover, a longer follow-up and the evaluation of both the ablation ratio and the retreatment rate would surely add interesting data to an evaluation such as ours. Anyway, we believe that our experience contributes to shedding light on a very promising topic, for which the literature is still quite sparse.

## Conclusion

In this retrospective, observational study, we found no substantial difference between ATN and FTN in the RFA of benign symptomatic thyroid nodules in terms of absolute volume reduction, VRR, success rate or safety over a 6-month follow-up. On the other hand, ATN was considered to be more straightforward and could consequently allow for shorter operator training and a wider and more efficient spreading of RFA as a natural alternative to surgery. However, these promising data need further validation, mainly with a longer follow-up and on the rate of eventual reintervention, before definitive conclusions can be drawn.

## Data Availability

The data that support the findings of this study are available on request from the corresponding author, M.R., upon reasonable request. The data are not publicly available due to privacy and ethical restrictions.
